# Type 1 Diabetes Mellitus Management and Islet Cell Therapy: A New Chapter in Patient Care

**DOI:** 10.7759/cureus.46912

**Published:** 2023-10-12

**Authors:** Sadaf Alam, Salman J Khan, Calvin Yee Fen Lee, Syed Asjad Tauheed Zaidi, Syeda Fatima Murtaza

**Affiliations:** 1 Internal Medicine, Meritus Health General Hospital, Hagerstown, USA; 2 Public Health, University of Massachusetts, Amherst, USA; 3 Hematology & Oncology, Mayo Clinic, Jacksonville, USA; 4 Internal Medicine, Jiangsu University, Zhenjiang, CHN; 5 Medicine, Shalamar Medical and Dental College, Lahore, PAK; 6 Medicine, Mayo Clinic, Jacksonville, USA; 7 Medicine, Allama Iqbal Medical College, Lahore, PAK

**Keywords:** severe hypoglycemia, insulin therapy, donislecel, islet cell therapy, type 1 diabetes mellitus (t1dm)

## Abstract

Diabetes mellitus (DM) is a complex endocrine disorder characterized by abnormally high levels of glucose, also called hyperglycemia. DM usually occurs when the body does not produce enough insulin or cannot respond to the insulin in the body. Type 1 diabetes mellitus (T1DM) or insulin-dependent diabetes is an autoimmune disease that affects around 8 million people in the world. Patients with T1DM experience an array of symptoms such as polyuria, polydipsia, and weight loss. These patients are prone to immediate life-threatening complications, including hypoglycemia and diabetic ketoacidosis. These patients are also at increased risk of ischemic heart disease, stroke, chronic kidney disease, vision loss, and even damage to nerve endings resulting in neuropathy. In this article, we will discuss type 1 diabetes mellitus and the different treatment options, focusing primarily on the Food and Drug Administration (FDA)-approved first cellular therapy for T1DM, donislecel.

## Editorial

Type 1 diabetes mellitus (T1DM) is one of the most common autoimmune diseases worldwide. In 2021, a study estimated around 8.4 million people living with T1DM worldwide. Of these individuals, 18% were under the age of 20 years, and 64% were between 20 and 59 years [1]. Moreover, T1DM is expected to affect around 13.5-17.4 million people by 2040 [1]. T1DM is an autoimmune disorder in which autoreactive T cells and autoantibodies, part of the patient’s immune system, destroy pancreatic beta cells responsible for producing insulin [2]. T1DM is diagnosed using glycated hemoglobin (HbA1c) and C-peptide levels [2]. A fasting blood glucose concentration above 126 mg/dL (7.0 mmol/L) and a random blood glucose concentration above 200 mg/dL (11.1 mmol/L) indicate DM. However, it must be noted that abnormal glycemia in the absence of symptoms must be recorded on two separate occasions. HbA1c is less reliable for diagnosing T1DM because of these patients' rapid progression of dysglycemia. Over 90% of those diagnosed with T1DM are estimated to have quantitative antibodies against specific beta-cell proteins, including insulin [2]. Two or more autoantibodies are associated with an 84% risk of T1DM by the age of 18 years. Studies have shown that T1DM is slightly more common in males than females [2].

There are a few treatment options for T1DM. However, injectable insulin remains the primary therapy for T1DM, with varying onsets and durations to optimize glycemic control. Most patients require a combination of rapid-acting insulin analogs, usually taken before meals, and long-acting insulin to maintain basal insulin levels, as this combination mimics the typical insulin pattern. Insulin is injected subcutaneously into the body through an insulin pen or insulin pump. Insulin must be adjusted according to the patient’s physical activity, food intake, chronic or acute illnesses, or stress. The only non-insulin medication approved for T1DM is pramlintide, used with mealtime insulin. It suppresses the glucagon level after meals and slows gastric emptying. It is used by less than 5% of the T1DM patients [2]. Some T1DM patients struggle with insulin treatment, leading to the development of major complications such as hypoglycemia and diabetic ketoacidosis. Hypoglycemia is considered the most common life-threatening complication, which is the cause of 4-10% of T1DM deaths [2].

To counter short-term life-threatening complications, researchers have been taking immense interest in therapies having the potential to cure T1DM by altering the immune system’s attack on beta cells. Since it is believed that T-cells play a role in attacking these beta cells, much of the therapy has been aimed at inhibiting T-cell activation. Trials of ciclosporin, an immunosuppressant, have been used to inhibit T-cell activation. Another form of cell therapy involving islet transplantation has been studied in patients with brittle T1DM with severe hypoglycemia. It involves islet beta-cell transplant, a relatively low-risk procedure where donor islet cells are transplanted into the liver via the portal vein [2].

Cell therapy has become a focus for the possible cure of T1DM. In June 2023, the Food and Drug Administration (FDA) approved the first cellular therapy, donislecel, for T1DM after clinical trials showed efficacy. A phase 1/2 clinical trial with 10 T1DM patients who had undergone cell transplants revealed that insulin independence was reached in 8 of 10 patients when fasting glucose levels were maintained below 140 mg/dL more than three times per week, and two-hour postprandial values were maintained below 180 mg/dL more than four times per week without the use of exogenous insulin. During the 15-month observation period, none of the 10 patients in either group presented with complete graft failure [3]. Sixty percent of the initially transplanted patients maintained insulin independence five years following the transplant [4]. Another phase-3 trial with 21 T1DM post-cell transplant patients showed significant glucose control and the absence of hypoglycemic episodes [5]. The trial revealed that 19 out of 21 patients maintained HbA1c ≤ 6.5% and reported the absence of severe hypoglycemic events at the one-year follow-up visit. It has been recommended that patients receiving this form of therapy need immunosuppressive medication to prevent rejection by their host immune system. The subjects were put on basiliximab, tacrolimus, sirolimus, etanercept, and exenatide [5].

There were no significant side effects reported for donislecel therapy. The main procedure-related adverse effect was bleeding. Medication-related events included weight loss, viral stomatitis, increased creatinine, proteinuria, transient anemia, nausea, and vomiting. Proteinuria is a known side effect of sirolimus. Replacing sirolimus with mycophenolate mofetil improved renal function. Follow-up studies showed a temporal trend of decreasing insulin independence, attributed to many factors like auto or allo-immune rejection, immunosuppressive drug toxicity, exhaustion, and the decline of islet cell mass [3,4].

T1DM puts lifelong dependence on continuous treatment and monitoring. Currently, insulin analogs are the main treatment options. However, in recent years, several trials working on curing T1DM revealed promising results for islet cell therapy. It led to the FDA approval of donislecel, the first such therapy that could potentially cure T1DM. The approval of this new therapy is a positive step forward, especially for those living with unmanageable brittle T1DM. It would also significantly reduce the hypoglycemic-related deaths in these patients.

## References

[1] Gregory GA, Robinson TIG, Linklater SE (2022). Global incidence, prevalence, and mortality of type 1 diabetes in 2021 with projection to 2040: a modelling study. Lancet Diabetes Endocrinol.

[2] DiMeglio LA, Evans-Molina C, Oram RA (2018). Type 1 diabetes. Lancet.

[3] Gangemi A, Salehi P, Hatipoglu B (2008). Islet transplantation for brittle type 1 diabetes: the UIC protocol. Am J Transplant.

[4] Qi M, Kinzer K, Danielson KK (2014). Five-year follow-up of patients with type 1 diabetes transplanted with allogeneic islets: the UIC experience. Acta Diabetol.

[5] (2023). Islet transplantation in type 1 diabetic patients using the University of Illinois at Chicago (UIC) protocol. https://classic.clinicaltrials.gov/ct2/show/study/NCT00679042?term=NCT03791567+%5BExpandedAccessNCTId%5D&draw=1&rank=1.

